# Biomimetic Nano‐Degrader Based CD47‐SIRPα Immune Checkpoint Inhibition Promotes Macrophage Efferocytosis for Cardiac Repair

**DOI:** 10.1002/advs.202306388

**Published:** 2024-03-13

**Authors:** Jinfeng Gao, Zhiqing Pang, Qiaozi Wang, Yiwen Tan, Qiyu Li, Haipeng Tan, Jing Chen, Wusiman Yakufu, Zhengmin Wang, Hongbo Yang, Jinyan Zhang, Dili Sun, Xueyi Weng, Qibing Wang, Juying Qian, Yanan Song, Zheyong Huang, Junbo Ge

**Affiliations:** ^1^ Department of Cardiology Zhongshan Hospital Fudan University Shanghai Institute of Cardiovascular Diseases Shanghai 20032 China; ^2^ National Clinical Research Center for Interventional Medicine Shanghai Clinical Research Center for Interventional Medicine 180 Feng Lin Road Shanghai 200032 China; ^3^ School of Pharmacy Fudan University Key Laboratory of Smart Drug Delivery Ministry of Education 826 Zhangheng Road Shanghai 200030 China; ^4^ Institute of Biomedical Sciences Fudan University Shanghai 20032 China

**Keywords:** acute myocardial infarction, CD47‐SIRPÎ± axis, efferocytosis, receptor‐mediated internalization, targeted protein degradation

## Abstract

CD47‐SIRPα axis is an immunotherapeutic target in tumor therapy. However, current monoclonal antibody targeting CD47‐SIRPα axis is associated with on‐target off‐tumor and antigen sink effects, which significantly limit its potential clinical application. Herein, a biomimetic nano‐degrader is developed to inhibit CD47‐SIRPα axis in a site‐specific manner through SIRPα degradation, and its efficacy in acute myocardial infarction (AMI) is evaluated. The nano‐degrader is constructed by hybridizing liposome with red blood cell (RBC) membrane (RLP), which mimics the CD47 density of senescent RBCs and possesses a natural high‐affinity binding capability to SIRPα on macrophages without signaling capacity. RLP would bind with SIRPα and induce its lysosomal degradation through receptor‐mediated endocytosis. To enhance its tissue specificity, Ly6G antibody conjugation (aRLP) is applied, enabling its attachment to neutrophils and accumulation within inflammatory sites. In the myocardial infarction model, aRLP accumulated in the infarcted myocardium blocks CD47‐SIRPα axis and subsequently promoted the efferocytosis of apoptotic cardiomyocytes by macrophage, improved heart repair. This nano‐degrader efficiently degraded SIRPα in lysosomes, providing a new strategy for immunotherapy with great clinical transformation potential.

## Graphical abstract

RBC membrane is hybridized with liposome (RLP) that dilutes its CD47 density to senescent RBC level and RLP is degraded with its receptor SIRPα in lysosomes through receptor‐mediated endocytosis, which further inhibits CD47‐SIRPα axis and increases the efferocytosis ability of macrophages.

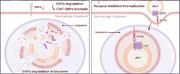

## 1 Introduction

As an innate immune checkpoint, the CD47‐SIRPα axis plays a critical role in the maintenance of tissue homeostasis and pathophysiological processes of various diseases, including tumor evasion, cardiovascular disease, and stem cell transplantation.^[^ ^1^ ^]^ It was first found to influence hemocyte maintenance as a “marker of self” for long circulation^[^ ^2^ ^]^ and was later revealed to be leveraged by malignant cells to evade immune phagocytosis by macrophages.^[^ ^3^ ^]^ Recently, pathophysiologically elevated CD47 and aberrant activation of CD47‐SIRPα were proved to be associated with atherosclerotic plaque vulnerability^[^ ^4^ ^]^ and adverse cardiac remodeling after acute myocardial infarction (AMI).^[^ ^5^ ^]^ Blocking this axis has been shown to encourage efficacy in tumor control and stem cell transplantation, as well as apoptotic cell efferocytosis in cardiovascular disease and fibrosis.^[^ ^4^, ^5^, ^6^ ^]^ Based on its key role in cell fate regulation, the CD47‐SIRPα axis may serve as a promising therapeutic target for multiple human diseases.

The current therapeutic approach targeting the CD47‐SIRPα axis primarily focuses on oncological indications and involves the use of monoclonal antibodies and fusion proteins. These interventions have demonstrated promising advancements in both preclinical and clinical studies.^[^ ^3^, ^7^ ^]^ However, direct administration of antibodies is limited by the occurrence of on‐target off‐tumor toxicity and antigen sinks.^[^ ^1a^ ^]^ Additionally, antibody‐based inhibitors are often associated with high costs, storage challenges, a heightened risk of immunogenicity, and a short half‐life in biological systems, which impede their widespread application. Therefore, there is an urgent need for the development of novel therapeutic strategies for blocking the CD47‐SIRPα immune checkpoint in a spatially specific manner.

Recently, targeted protein degradation (TPD), which harnesses the natural protein degradation machinery, including the ubiquitin‐proteasome system^[^ ^8^ ^]^ and the lysosomal degradation pathway^[^ ^9^ ^]^ to selectively target and eliminate disease‐related proteins, has achieved great advances in drug discovery and has shown promising activity in cancer therapy.^[^ ^10^ ^]^ Among them, lysosome‐targeted chimeras (LYTACs) utilize the endocytosis lysosomal pathway to degrade target proteins, particularly focusing on membrane proteins and extracellular proteins, which holds great promise for advancing CD47‐SIRPα checkpoint inhibition. Typically, LYTACs consist of antibodies that can simultaneously bind to lysosomal targeting receptors (LTRs) on the cell surface and extracellular or transmembrane proteins. Following the recognition of proteins of interest (POI) by antibodies, LYTACs form a ternary complex with LTRs on the cell surface, facilitating the transportation of POI to the lysosomes for degradation.^[^ ^10^ ^]^ Although TPD has shown favorable attributes in both preclinical and clinical settings, there are still some hurdles that restrict its clinical application, including inadequate tissue penetration, poor stability, and poor cell targeting.^[^ ^11^ ^]^ However, the precise mechanism underlying the recruitment of components associated with the lysosomal pathway remains unclear, and the intricate structure of LYTACs requires further optimization. Therefore, the development of a novel TPD strategy to improve the tissue targeting capability is necessary for the effective implementation of CD47‐SIRPα checkpoint inhibition.

Nanomaterials have attracted significant attention in the field of disease treatment as carriers^[^ ^12^ ^]^ or for demonstrating drug‐like properties.^[^ ^13^ ^]^ When certain nanoparticles interact with receptor cells, they enter the cells through receptor‐mediated endocytosis and further enter lysosomes for degradation, which may serve as a unique LYTAC to mediate protein degradation. Simultaneously, nanoparticles also exhibit favorable flexibility, enabling the incorporation of targeted molecules for specific cells and ligand molecules for binding to target proteins,^[^ ^14^ ^]^ which may enhance the efficacy of TPD drug targeting. Previous studies have shown that nanomaterials can trigger the internalization and degradation of surface proteins within lysosomes,^[^ ^15^ ^]^ showing great potential for eliminating therapeutically relevant proteins. Based on these findings, we speculate that nanomaterials may present a novel avenue for TPD, as the nanoparticles themselves can serve as lysosome‐targeting elements. When nanoparticles are modified with molecules capable of binding to POI, they can engage in the cellular phagocytosis pathway and undergo endocytosis alongside the POI, further promoting POI degradation in lysosomes, acting as LYTAC‐like agents, while avoiding the tedious process of screening for suitable LTRs. Moreover, nanomaterials can be modified with moieties to target specific tissues or cells to enhance their targeting properties.^[^ ^16^ ^]^ Therefore, the utilization of nanomaterials presents a novel approach for advancing TPD technology and facilitating the suppression of the CD47‐SIRPα immune checkpoint.

**Figure 1:**
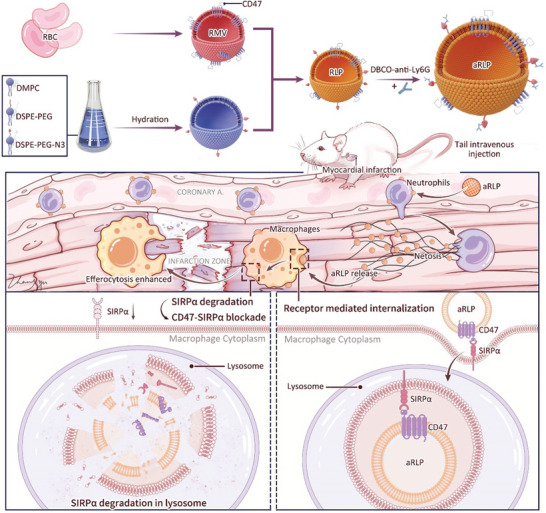
Schematic diagram of aRLP fabrication, which served as a nano‐degrader to block the CD47‐SIRPα axis and promote macrophage efferocytosis.

In this study, we introduced a biomimetic liposome as a nano‐degrader to degrade SIRPα on macrophage membrane and tested the effect on inhibition of CD47‐SIRPα immune checkpoint in myocardial infarction as the disease model. The efficacy of the TPD is primarily contingent on its ability to selectively bind to POI. Biomimetic nanosystems employ natural ligands for target receptors, frequently from the corresponding cell membrane, to modify nanomaterials, thereby inheriting the characteristics of the cell and possessing higher affinity and specificity for the target protein.^[^ ^17^ ^]^ Indeed, CD47 molecule was initially discovered to be highly expressed on the surface of red blood cells (RBC) for immune privilege, which renders it an ideal candidate as a natural ligand for SIRPα with high affinity. During RBC aging, a decline in cell surface CD47 expression was observed and was shown to promote the clearance of senescent RBCs,^[^ ^18^ ^]^ which tend to be removed from the lysosomes of macrophages for iron recycling.^[^ ^19^ ^]^ Notably, RBCs with a 50% loss of CD47 in CD47^+/‐^ mice are more susceptible to clearance by macrophages.^[^ ^20^ ^]^ These findings suggested that CD47 could bind to SIRPα with high affinity as the naïve ligand, while the innate activation effect of CD47 on CD47‐SIRPα axis acted in a density‐dependent manner which illustrated that as the expression density of CD47 decreased, their interaction with SIRPα was insufficient to induce the clustering of SIRPα and activation of CD47‐SIRPα axis. Here, we introduced a senescent RBC‐mimetic liposome using cell membrane hybridized technology to simulate decreased CD47 density and function as a novel nano‐degrader for SIRPα (**Figure** **1**). Senescent RBC‐mimetic liposomes (RLP) were fabricated by fusing RBC membrane vesicles (RMV) with liposomes to dilute CD47 density to the senescent level. RLP retained the binding ability with SIRPα while with little downstream activation effect. Upon transport to macrophages, CD47 would mediate the interaction of RLP with SIRPα and subsequent internalization into lysosomes. To overcome the poor innate targeting ability and compromised immune escape ability of RLP after hybridization, we decorated RLP with an anti‐Ly6G antibody (aRLP) to hitch neutrophils (NEs) in circulation, which are widely used as drug carriers in inflammatory environments.^[^ ^21^ ^]^ NEs, the first responsive and largest population of myeloid cells, are mobilized and recruited to infarcted and border areas in the first few hours after AMI.^[^ ^22^ ^]^ Upon reaching the site of inflammation, NEs would release their cargo through the formation of neutrophil extracellular traps (NETs). After extensive characterization and exploration of aRLP, the SIRPα degradation efficiency of aRLP was tested in vitro. Furthermore, in a murine model of AMI, aRLP would hijack circulating neutrophils and transport them into the infracted myocardium where they acted as a LYTAC‐like agent to mediate the internalization and degradation of SIRPα in lysosomes of macrophages and consequently inhibited the CD47‐SIRPα axis. This process enhances the efferocytosis of apoptotic cardiomyocytes (CMs) and improves cardiac repair. Through the utilization of biomimetic nanotechnology, this study represents a novel approach for the development of nano‐degraders based on TPD strategy and immunotherapy for CD47‐SIRPα immune checkpoint inhibition.

## 2 Results

### 2.1 Senescent RBC‐Mimetic RLP Fabrication and SIRPα Degradation

**Figure 2:**
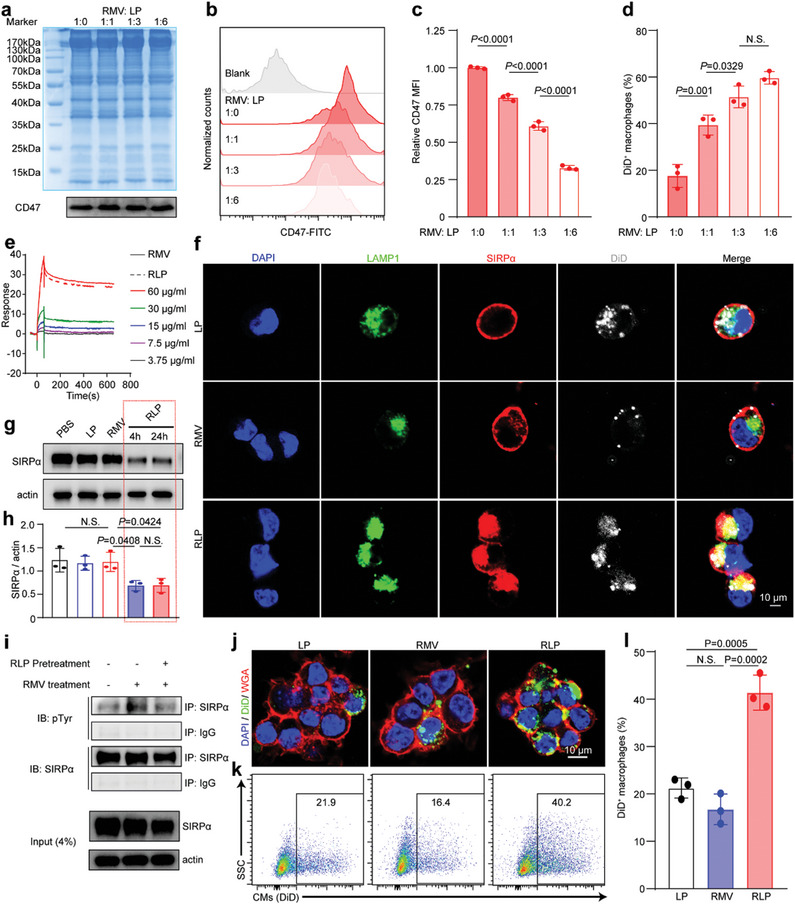
Senescent RBC‐mimetic RLP fabrication and SIRPα degradation. a) Coomassie blue staining and CD47 western blotting of 1:0, 1:1, 1:3, and 1:6 of RMV: LP (mass ratio). b) Flow cytometry analyses of CD47 MFI of 1:0, 1:1, 1:3, and 1:6 of RMV: LP and quantification (c). Statistical analysis was calculated using the one‐way ANOVA and Tukey's tests (*n* = 3). d) Flow cytometry analyses of macrophages internalization of 1:0, 1:1, 1:3, 1:6 of RMV: LP particles. Statistical analysis was calculated using the one‐way ANOVA and Tukey's tests (*n* = 3). e) Surface plasmon resonance of RMV or RLP binding to immobilized macrophages. f) Immunostaining to show the location of SIRPα in macrophages 1 h after treatment with LP, RMV, and RLP. g) Expression level of SIRPα by western blot assay 4 h after treatment with PBS, LP, RMV, RLP, and RLP (24 h) and quantification (h). Statistical analysis was calculated using the one‐way ANOVA and Tukey's tests (*n* = 3). i) Immunoprecipitation to detect the SIRPα phosphorylation level of macrophages without treatment, with RMV treatment, or RMV treatment with pre‐treatment of RLP. j) Representative images of macrophage immunostaining with LP, RLP, or aRLP pre‐treatment to engulf DiD‐labeled apoptotic CMs and corresponding flow cytometry (k) and quantification (l). Statistical analysis was calculated using the one‐way ANOVA and Tukey's tests (*n* = 3).

RBCs were extracted from healthy mice, and RMV were obtained using the hypotonic method^[^ ^13a^ ^]^ followed by centrifugation. Liposomes (LP) were prepared using TFH. After RMV derivation, we first verified that the amount of CD47 protein in the RBC was not significantly reduced (Figure S1, Supporting Information), indicating that the CD47 density did not change on RMV compared to that on RBC. A previous study using CD47^+/−^ mice revealed that a 50% loss of CD47 rendered RBCs a senescent phenotype that was more easily cleared by macrophages.^[^ ^20^ ^]^ Through the calculation of the surface area of RBC and liposomes (see Supporting Information), we found the fusion of 0.3 mg protein RMV with 0.9 mg LP would gain a theoretic 50% CD47 density nanoparticle. To verify the senescent nature of nanoparticles, we constructed a series of CD47 density gradient nanoparticles by hybridizing a fixed amount of 0.3 mg protein RMV with LP in mass ratios of 1:1, 1:3, and 1:6, resulting in predicted CD47 densities of 75%, 50%, and 33%, respectively. Coomassie blue staining and western blotting showed that the protein composition and CD47 content profiles were similar among the four nanoparticles (**Figure** **2**a). Further characterization and functional comparison showed that these nanoparticles exhibited comparable diameters and polydispersity indices (PDI), albeit with varying zeta potentials (Table S1, Supporting Information). We also found that CD47 positive particles were all more than 93% by flow cytometry (Figure S2, Supporting Information), indicating satisfactory membrane hybridization. Importantly, we obtained the CD47 mean fluorescence intensity (MFI) of these four nanoparticles to determine their relative CD47 density. By standardizing RMV as 100% in CD47 density as RBC, the CD47 density of 1:1, 1:3 and 1:6 ratio particles were 79.97 ± 2.03%, 60.81 ± 2.94%, and 32.87 ± 1.48%, respectively (Figure 2b,c).

Because senescent RBC is prone to phagocytosis by macrophages due to a decreased level of surface CD47,^[^ ^18^ ^]^ we incubated RMV and RLP of different CD47 densities with macrophages for 1 h to detect the effect of CD47 density on the phagocytosis of nanoparticles. With higher CD47 decoration, RMV showed significantly less phagocytosis than the other hybridized particles. Phagocytosis efficiency was inversely related to CD47 density, with a plateau phase reached when the phagocytosis of the 1:6 particles was similar to that of the 1:3 particles (Figure 2d; Figure S3, Supporting Information). These results showed that macrophage phagocytosis depended on the density of CD47 on nanoparticles, and RLP with reduced CD47 density could simulate the phagocytosis of senescent RBCs by macrophages. Because there was a plateau phase, we chose RLP from a 1:3 ratio with ≈60% CD47 density as our nano‐degrader for the following experiments.

Next, the targeted degradation of SIRPα by RLP was investigated in vitro. Macrophages were treated with RLP for 20 min, 1 h, and 4 h, and dynamic changes in binding, internalization, 1 h, and degradation were observed. First, we investigated the binding affinity of RLP to the SIRPα by surface plasmon resonance (SPR) and confocal microscope. SPR showed that RLP bound to macrophages with a K_a_ of (2.37 ± 0.17) × 10^2^ (mg mL)^−1^ s^−1^ and *K*_d_ of 0.42 ± 0.03 µg mL^−1^ s^−1^ and RMV bound to macrophages with a *K*_a_ of (2.67 ± 0.18) × 10^2^ (mg mL)^−1^ s^−1^ and *K*_d_ of 0.37 ± 0.03 µg mL^−1^ s^−1^, indicating that RLP and RMV hold similar binding ability with macrophages (Figure 2e). After treatment for 20 min, the cells were gently fixed and visualized by confocal microscopy. The application of RMV treatment resulted in a significant increase in the accumulation of SIRPα at the binding site, whereas the clustering of SIRPα was not observed in response to RLP or LP treatment. (Figure S4, Supporting Information). Given that SIRPα clustering was previously deemed crucial for the activation of the CD47‐SIRPα axis, these findings suggest that RLP possessed the ability to bind to SIRPα with its inherent affinity, even in the presence of reduced CD47 expression, without triggering SIRPα activation. Further immunoprecipitation assay demonstrated the same result, for RLP would not increase the phosphorylation level of SIRPα (Figure S5, Supporting Information). Given that nanoparticles binding with receptors could induce its internalization,^[^ ^23^ ^]^ we speculated that SIRPα could be endocytosed along with RLP by macrophages. To trace SIRPα after internalization before degradation, macrophages were fixed after treatment with DiD labeled LP, RMV, and RLP for 1 h and stained with SIRPα and lysosome‐associated membrane protein 1 (LAMP1). Confocal imaging revealed the intracellular colocalization of RLP, SIRPα, and LAMP1, while LP was directly internalized to the lysosome and little RMV was internalized. These findings suggested that only RLP, which mimicked senescent RBC, could promote the internalization of SIRPα into lysosomes (Figure 2f). Receptor‐mediated endocytosis was mostly performed through the clathrin or caveolin pathway^[^ ^24^ ^]^ and we used chlorpromazine (clathrin‐mediated endocytosis inhibitor) and filipin (caveolin‐mediated endocytosis inhibitor) to investigate the mechanism and the role of endocytosis on the lysosomal localization of SIRPα. The result showed that filipin significantly inhibited SIRPα endocytosis while chlorpromazine had little effect, suggesting that SIRPα‐mediated RLP internalization was mainly through caveolin pathway after CD47‐SIRPα interaction and cell membrane invagination (Figure S6, Supporting Information). Then the degradation efficiency of SIRPα was investigated by western blotting after treatment for 4 h. SIRPα was significantly degraded by RLP treatment and this degradation effect could last for 24 h (Figure 2g,h). These results illustrated that RLP could trigger the internalization of SIRPα into lysosomes where the targeted protein would be further degraded.

Then we explored whether SIRPα degradation on macrophages would benefit CD47‐SIRPα axis blocking. We washed macrophages twice and RMV was added as a SIRPα agonist 4 h after pre‐treatment with RLP. Untreated macrophages served as a baseline control, and RMV treatment served as a positive control. Consequently, the SIRPα phosphorylation level was lower when pre‐treated with RLP than positive control (Figure 2i). Finally, we tested the efferocytosis levels of apoptotic CMs in macrophages pretreated with RLP in vitro. Immunostaining revealed that RLP‐pretreated macrophages engulfed more apoptotic CMs than LP and RMV controls (Figure 2j). Flow cytometry also showed improved macrophage efferocytosis in apoptotic CMs treated with RLP (Figure 2k,l). Furthermore, we measured whether enhanced efferocytosis would promote macrophages to be in an M2 state and to secret anti‐inflammatory cytokines. Western blotting and flow cytometry showed elevated CD206 expression in the RLP group compared with that in the LP and RMV groups, which was a typical M2 marker (Figure S7, Supporting Information). In the supernatant, though pro‐inflammatory cytokines like IL‐1β, IL‐6, and TNF‐α showed no significant changes, anti‐inflammatory cytokines like IL‐10 and TGF‐β were all higher in the RLP group (Figure S8, Supporting Information). Taken together, RLP could bind with SIRPα with high affinity via mimicking senescent RBC and served as a nano‐degrader to mediate the internalization and degradation of SIRPα in lysosomes, thus blocking the CD47‐SIRPα axis and promoting the efferocytosis of apoptotic CMs by macrophages, which further shift macrophages to an anti‐inflammatory state.

### 2.2 Conjugation of Ly6G Antibody to RLP and Characterization

**Figure 3:**
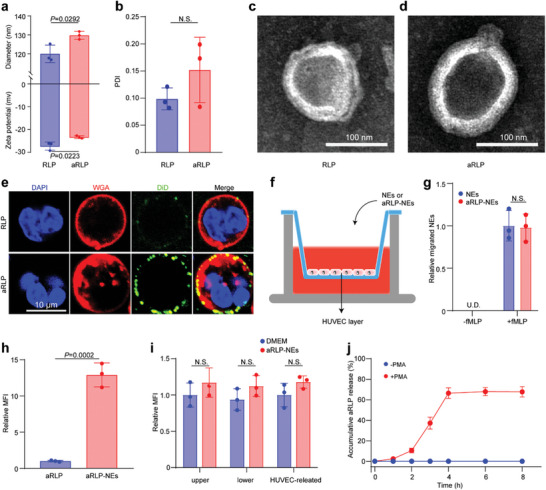
aRLP characterization and aRLP‐NEs function identification. Diameter, zeta potential (a), and PDI (b) of RLP and aRLP. Statistical analysis was calculated using the two‐sided Student's *t*‐test (*n* = 3). TEM images of RLP (c) and aRLP (d). e) Representative images of immunostaining of NEs incubated with DiD labeled RLP or aRLP for 15 min. f) Schematic illustration of the aRLP‐NEs migration model. g) Relative migrated the number of NEs and aRLP‐NEs in the lower chamber with or without 10 nM fMLP. U.D., undetected. Statistical analysis was calculated using the two‐sided Student's *t*‐test (*n* = 3). h) Relative MFI of aRLP in the lower chamber by adding free aRLP or aRLP‐NEs to the upper chamber. Statistical analysis was calculated using the two‐sided Student's *t*‐test (*n* = 3). i) relative MFI of aRLP in the upper chamber, lower chamber, and HUVEC layer. Statistical analysis was calculated using the two‐sided Student's *t*‐test (*n* = 3). j) Release profile of aRLP from aRLP‐NEs with or without treatment of 100 nM PMA.

Owing to the lowered CD47 density, senescent RBC‐mimetic RLP has short circulating periods in the blood and insufficient accumulation in the heart, which allowed us to utilize the neutrophil hitchbike strategy to increase the targeting ability of RLP. Ly6G is a specific surface marker of mouse NEs^[^ ^25^ ^]^ and the Ly6G antibody was conjugated with RLP via click chemistry to hitchhike NEs. First, the DBCO modification of Ly6G was verified using ultraviolet spectroscopy (Figure S9, Supporting Information), and DBCO‐Ly6G was conjugated to the azide group on the RLP (aRLP). The diameter of aRLP increased from 120.0 ± 4.6 nm of RLP to 129.7 ± 2.1 nm and the zeta potential was neutralized from −27.27 ± 1.76 to −23.37 ± 0.61 mv due to the positive charge of Ly6G antibody in the neutral environment (**Figure** **3**a). The PDI of the RLP and aRLP were similar (Figure 3b). Immunostaining of aRLP showed co‐localization of RLP (green) with the Ly6G antibody (red), indicating successful conjugation of the Ly6G antibody with RLP (Figure S10, Supporting Information). Transmission electron microscopy (TEM) showed no significant morphological differences between RLP and aRLP (Figure 3c,d).

Primary NEs were isolated from mouse bone marrow, and their purity was identified by flow cytometry (Figure S11, Supporting Information), which was subsequently used to observe the benefit of the Ly6G antibody. RLP or aRLP was incubated with NEs for 15 min, and immunostaining showed that aRLP gathered on the membrane of NEs more than RLP (Figure 3e), indicating that aRLP could hijack NEs with a high affinity. Next, we investigated the function of NEs after aRLP binding. Cell viability was not affected by nanoparticle binding (Figure S12, Supporting Information). A Transwell assay was performed to test whether the chemotaxis of NEs was compromised by aRLP binding (Figure 3f). Without formyl methionyl‐leucyl‐phenylalanine (fMLP) treatment, NEs or aRLP‐NEs were not detected in the lower chamber, suggesting the successful establishment of an endothelial barrier. The addition of 10 nM fMLP to the lower chamber sharply increased the number of NEs that migrated across the endothelial barrier; however, there was no obvious difference in the number of NEs or aRLP‐NEs (Figure 3g). aRLP were designed for targeted therapy with the help of circulating NEs, and we found that free aRLP could not be transported to the lower chamber, whereas aRLP‐NEs accumulated much more (Figure 3h). During NEs migration, we also did not observe apparent detachment of DiD‐labeled aRLP from NEs in the compartments of the upper chamber, lower chamber, and HUVEC layer (Figure 3i). Upon stimulation with phorbol myristate acetate (PMA), NEs would form NETs and release their cargo. aRLP was released in the first 4 h and the final release amount was 67.8 ± 5.1% (Figure 3j). However, aRLP was almost unreleased in the absence of PMA, indicating a relatively stable conjugation. These data suggest that NEs binding to aRLP preserves the function of chemotaxis without premature detachment and releases cargos at the targeted site.

### 2.3 Selective Hitchhiking of aRLP in Neutrophils and Targeted Release

**Figure 4:**
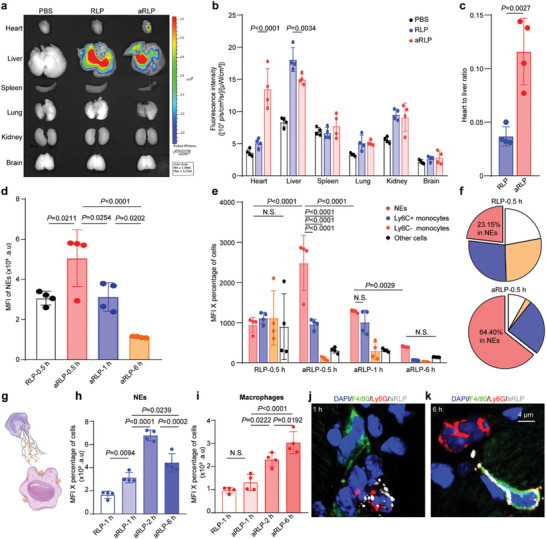
Heart targeting of aRLP by NEs hitchhiking and release. a) Presentative images of major organs by ex vivo IVIS in PBS, RLP, aRLP group 2 h after injection and quantification (b). Data were analyzed by average radiant efficiency. Statistical analysis was calculated using the two‐way ANOVA and Tukey's multiple comparison tests. c) Heart‐to‐liver ratio of RLP and aRLP group by dividing the total radiant efficiency of the heart to liver. Statistical analysis was calculated using the two‐sided Student's *t*‐test (*n* = 4). d) Peripheral blood flow cytometry analyses of cellular DiD MFI of NEs from the RLP group (0.5 h) and aRLP group (0.5, 1, and 6 h). Statistical analysis was calculated using the one‐way ANOVA and Tukey's tests (*n* = 4). e) Relative distribution of nanoparticles in blood cells from the RLP group (0.5 h) and aRLP group (0.5, 1, and 6 h) calculated by the equation (ΣMFI_X_ × Percentage_X_)/Σ(MFI_X_ × Percentage_X_). X represents blood cells including NEs, Ly6C+ monocytes, Ly6C− monocytes, or other cells. Statistical analysis was calculated using the two‐way ANOVA and Tukey's multiple comparison tests. f) Pie graph emphasizing relative distribution of nanoparticles in blood cells from RLP group and aRLP group 0.5 h after injection. Relative nanoparticle accumulation in NEs (h) and macrophages (i) of hearts from the RLP group (1 h) and aRLP group (1, 2, and 6 h) was calculated by the equation (MFI_X_ × Percentage_X_). X represents NEs or macrophages. Statistical analysis was calculated using the one‐way ANOVA and Tukey's tests (*n* = 4). aRLP (white) transfer observation by immunostaining of NEs (red) and macrophages (green) in hearts 1 h (j) and 6 h (k) after injection.

Next, we evaluated the in vivo biodistribution of aRLP and explored the mechanisms underlying targeted heart delivery. After AMI, NEs were elevated within 2 h and peaked at the 6‐h point,^[^ ^22^ ^]^ which largely mobilized to the heart. To maximize the loading capacity of NEs, the nanoparticles were injected into mice via the tail vein 6 h post‐AMI. Major organ imaging 2 h after injection using an in vivo spectrum imaging system showed that Ly6G antibody‐decorated nanoparticles accumulated in infarcted hearts ≈2.7‐fold more than RLP (**Figure** **4**a,b), mainly in the infarct and border areas of the left ventricle. Notably, considering liver clearance, we calculated the heart‐to‐liver ratio and found that it increased 3‐fold between the aRLP and RLP groups (Figure 4c).

**Figure 5:**
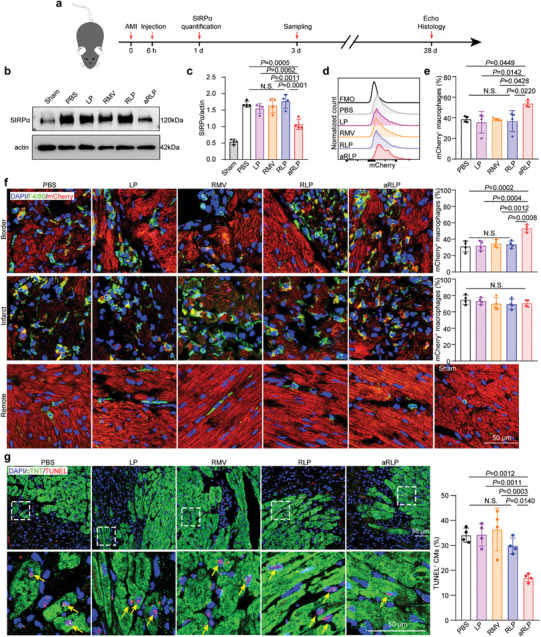
aRLP mediated SIRPα degradation and efferocytosis promotion. a) The in vivo experimental design timeline. b) SIRPα level 18 h after PBS, aLP, aRMV, RLP, and aRLP injection by western blot assay and quantification (c). d) Statistical analysis was calculated using the one‐way ANOVA and Tukey's tests (*n* = 4). e) Flow cytometry analysis of mCherry positive macrophages 3 d after AMI and quantification. Statistical analysis was calculated using the one‐way ANOVA and Tukey's tests (*n* = 4). f) Representative images of F4/80 (green) immunostaining indicating mCherry (red) positive CMs engulfed by macrophages from hearts of border, infarct, and remote area and quantification of border and infarct area. Statistical analysis was calculated using the one‐way ANOVA and Tukey's tests (*n* = 4). g) Representative CMs apoptosis images of cTnT and TUNEL co‐immunostaining and quantification. Yellow arrowheads indicate apoptotic CMs. Statistical analysis was calculated using the one‐way ANOVA and Tukey's tests (*n* = 4).

To further explore the role of NEs in nanoparticle delivery, we performed flow cytometry on the peripheral blood. 0.5 h after injection, a significant increase in the MFI of NEs was observed in the aRLP group (Figure 4d). After 1 and 6 h, the MFI of the NEs decreased. Specifically, we calculated the relative distribution of the nanoparticles in blood cells. In aRLP group, 64.4% of nanoparticles were distributed in NEs, much higher than that in the RLP group (23.15%) 0.5 h after injection (Figure 4e,f). In monocytes, no difference was observed, indicating that monocytes did not contribute to nanoparticle targeting. We also delineated the dynamic profile of aRLP in all blood cells at 0.5, 1, and 6 h after injection and found a gradual decrease in fluorescence intensity in NEs (**Figure** **5**d,e), mirroring that NEs binding with aRLP were migrated to the heart. Together, these results revealed the selectivity of aRLP in hitchhiking neutrophils.

We then turned our focus from blood to the heart. At the target site, NEs would form NETs and concurrently release nanoparticles^[^ ^21^ ^]^ (Figure 5g). In view of the traveling time of NEs, we performed flow cytometry of the hearts 1, 2, and 6 h after injection. We found that the percentage of NEs infiltrating the heart was not influenced in vivo 1 h after injection in the PBS, RLP, or aRLP groups (Figure S13, Supporting Information), confirming that the chemotaxis of NEs was not impaired by aRLP binding. We found that the fluorescence intensity of aRLP reaching hearts infarcted by NEs was much higher than that of RLP (Figure 4h). Interestingly, in the aRLP group, we found an increase in the fluorescence intensity of NEs from 1 to 2 h and a decrease at 6 h, suggesting the overall accumulation of nanoparticles carried by NEs at the first and later release. Meanwhile, in macrophages, there was no difference in the fluorescence of the nanoparticles 1 h after injection, indicating that macrophages contributed little to aRLP delivery. However, aRLP in macrophages increased continuously from 2 to 6 h, corresponding to a decrease in aRLP in NEs (Figure 4i). At the cellular level, we performed immunostaining of NEs and macrophages to determine the location of aRLP at 1 and 6 h. At 1 h, aRLP gathered around the NEs (Figure 4j) and was transferred to neighboring macrophages at 6 h (Figure 4k). In summary, these results indicate that NEs carry aRLP into the ischemic myocardium and release it into macrophages, which is necessary for further treatment.

### 2.4 SIRPα Degradation and Apoptotic CMs Efferocytosis Promotion by aRLP

To evaluate the therapeutic effects evaluation, we designed experiments in vivo as the timeline of Figure 5a. The mice were randomly distributed into Sham, PBS, LP, RMV, RLP, and aRLP groups. Benefitting from the excellent targeting ability, we found that SIRPα was significantly decreased after aRLP injection (Figure 5b,c). MYH6 (Myosin Heavy Chain 6) promoter‐driven mCherry transgenic mice were used to label and trace apoptotic CMs and investigate the efferocytosis ability of macrophages. Flow cytometry suggested improved apoptotic CMs clearance by increasing the percentage of mCherry‐positive macrophages in the aRLP group compared with that in the other groups (Figure 5d,e). Specifically, confocal imaging showed that the percentage of mCherry‐positive macrophages in the border area increased after aRLP injection (Figure 5f, top). Furthermore, we noticed that in the infarct area, the percentage of mCherry‐positive macrophages was similar in all groups (Figure 5f, mid), indicating that nanoparticle internalization did not influence the macrophages to ingest debris from necrotic CMs. Moreover, we also explored whether decreased SIRPα would induce macrophages to attack viable CMs. There were fewer macrophages in the remote area, and almost no mCherry signals were engulfed, as aRLP mainly accumulated in the myocardial ischemic area (Figure 5f bottom; Figure S14, Supporting Information). In addition, we found that, in the border area, free apoptotic CMs were lower in the aRLP group than in the other groups (Figure 5g). These results indicated that aRLP could enhance apoptotic CMs efferocytosis through SIRPα degradation without affecting debris elimination or normal CMs phagocytosis.

### 2.5 Decreased Infarct Size and Reparatory Microenvironment Elicited by aRLP

**Figure 6:**
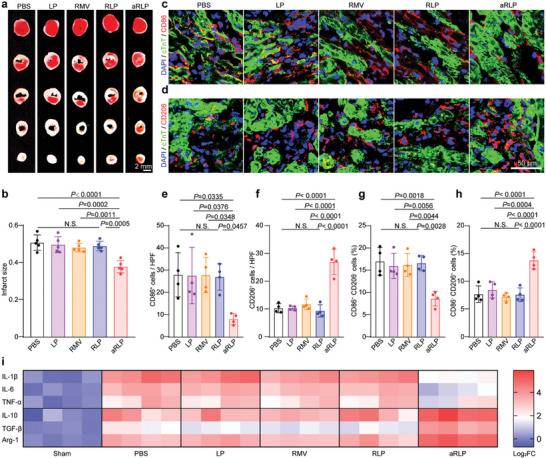
Decreased infarct size and reparatory microenvironment elicited by aRLP. a) Infarct size determination by TTC staining in PBS, aLP, aRMV, RLP, aRLP groups, and quantification (b). Statistical analysis was calculated using the one‐way ANOVA and Tukey's tests (*n* = 5). Representative immunostaining images of cTnT and CD86 (c) or CD206 (d) in the border area of infarcted hearts from PBS, RLP, aRLP groups and quantification (e, f). Statistical analysis was calculated using the one‐way ANOVA and Tukey's tests (*n* = 4). Flow cytometry analysis of infiltrated leukocytes (g) and macrophage phenotyping (h). CD86^+^ CD206^−^ population represents M1‐like pro‐inflammatory macrophages and CD86^−^ CD206^+^ population represents M2‐like anti‐inflammatory macrophages. i) qPCR analysis indicating the transition of pro‐inflammatory microenvironment to anti‐inflammatory microenvironment by heatmap. Statistical analysis was calculated using the one‐way ANOVA and Tukey's tests (*n* = 4).

We investigated whether efferocytosis enhancement by aRLP could improve the early repair of AMI. 2,3,5‐Triphenyltetrazolium chloride (TTC) staining indicated that the mice injected with aRLP had smaller infarct sizes than those in the other groups (**Figure** **6**a,b). Mechanistically, enhanced efferocytosis by aRLP resulted in decreased CD86^+^ pro‐inflammatory macrophages and increased CD206^+^ anti‐inflammatory macrophages in the border area (Figure 6c–f), which were further quantified by flow cytometry (Figure 6g,h; Figure S15, Supporting Information). We used reverse transcription‐quantitative polymerase chain reaction (RT‐qPCR) to measure the expression of typical inflammation‐related genes. Pro‐inflammatory genes including IL‐1β, IL‐6, and TNF‐α were down‐regulated and anti‐inflammatory genes including IL10, TGF‐β, and Arg‐1 were up‐regulated in aRLP group (Figure 6i). Correspondingly, pro‐inflammatory cytokines including IL‐1β, IL‐6, and TNF‐α were down‐regulated and anti‐inflammatory cytokines including IL10 and TGF‐β were up‐regulated in the heart with aRLP treatment (Figure S16, Supporting Information). Together, these results suggested that inhibition of CD47‐SIRPα immune checkpoint enhanced efferocytosis, decreased infarct size, and elicited a reparatory microenvironment.

### 2.6 Improved Cardiac Function and Remodeling by aRLP

**Figure 7:**
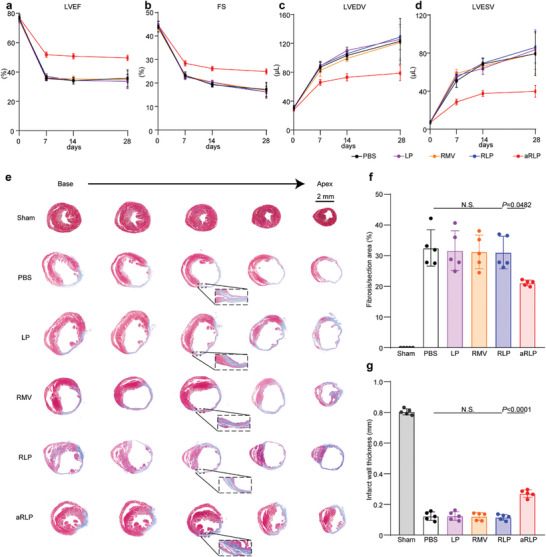
Improved cardiac function and remodeling by aRLP. a–d) Echocardiography measurements of LVEF, FS, LVEDV, and LVESV of Sham mice or AMI mice injected with PBS, RLP, and aRLP (*n* = 5). Statistical analysis was calculated using the one‐way ANOVA and Tukey's tests. e) Representative images of Masson's trichrome staining of mice hearts from Sham mice or AMI mice injected with PBS, RLP, and aRLP. f,g) Quantification of cardiac fibrosis area and infarct wall thickness (*n* = 5). Statistical analysis was calculated using the one‐way ANOVA and Tukey's tests.

To investigate whether enhanced efferocytosis translated into a long‐term therapeutic prognosis, we evaluated cardiac function and remodeling before 7, 14, and 28 days after AMI. Echocardiography showed increased left ventricular ejection fraction (LVEF) and fraction shortening (FS) in mice injected with aRLP compared to those in the PBS group, whereas the LP, RMV, and RLP groups showed no significant improvement (**Figure** **7**a,b). Similarly, the aRLP group had lower left ventricular end‐diastolic volume (LVEDV) and left ventricular end‐systolic volume (LVESV) than the other groups (Figure 7c,d). With respect to cardiac remodeling, Masson's trichrome staining revealed a smaller fibrotic area in the aRLP group, particularly in the border zone of the heart (Figure 7e,f). Additionally, we observed a thicker left ventricular infarct wall in the hearts of the aRLP group (Figure 7g). These results indicated that aRLP could effectively improve cardiac function in MI mice by SIRPα degradation‐induced CD47‐SIRPα axis inhibition.

### 2.7 Biocompatibility of aRLP

Finally, we evaluated the biocompatibility of aRLP in healthy mice. Compared with PBS injection, the concentration of IL‐1β and TNF‐α in plasma showed no significant elevation 6 h after aRLP injection (Figure S17a,b, Supporting Information). Moreover, we did not find obvious morphological changes in major organs, including the liver, spleen, lung, kidney, and brain, 7 days after PBS or aRLP injection (Figure S17c, Supporting Information). Taken together, these results indicate that aRLP is safe in mouse models.

## 3 Discussion

CD47‐SIRPα axis played an important role in physiological tissue homeostasis and served as a promising therapeutic target in various diseases.^[^ ^1b^ ^]^ Targeted protein degradation which utilizes the inherent intracellular proteolysis systems to target specific proteins for degradation has emerged as a powerful new drug discovery strategy^[^ ^11^ ^]^ and provided a new therapeutic strategy to inhibit CD47‐SIRPα axis. In this study, we demonstrated that senescent RLP served as a nano‐degrader, acting as a LYTAC‐like agent, to inhibit CD47‐SIRPα axis through SIRPα degradation, which further led to enhanced macrophage efferocytosis and improved cardiac function after AMI.

Traditional LYTACs typically comprise three components: a ligand targeting the POI, a ligand targeting the LTR, and a linker connecting the two moieties.^[^ ^26^ ^]^ In this study, we utilized the natural ligand CD47 molecule to target SIRPα protein, and further simulated the CD47 density distribution on senescent RBC by membrane hybridization to disable the SIRPα downstream pathway activation by CD47. SPR assay and confocal microscopic imaging revealed that the binding ability of CD47 on RLP to SIRPα was similar to that on RMV, while RLP showed little SIRPα clustering and phosphorylation, indicating that RLP could bind with SIRPα without activation of CD47‐SIRPα axis. Here liposomes served as moiety to guide SIRPα protein into lysosome. After CD47 binding with SIRPα, RLP entered the cells by receptor‐mediated endocytosis and carried SIRPα protein to lysosomes for degradation. Endocytosis is one of the main mechanisms through which liposomes interact with cells. After phagocytosis, the cell membrane collapses to form endosomes, which further fuse with lysosomes to degrade the liposomes. Using this natural metabolic pathway, liposomes could function as ligands that target LTR by directing POI to lysosomes, avoiding the tedious process of screening the related recruiting elements of the lysosome pathway, and greatly improving protein degradation efficiency. Liposomes also provide a platform to connect other targeted elements because of their easy modification to improve the tissue penetration and cell selectivity of the drug, which greatly hinders TPD's clinical application of TPD.

To enhance the accumulation of RLP in the target tissue, the conjugation of Ly6G antibody to RLP was employed and facilitated the attachment of aRLP to circulating neutrophils, thereby augmenting the targeting capability of aRLP toward the infarcted heart and subsequent SIRPα degradation effect. Their natural homing property to inflammatory sites renders neutrophils optimally targeted delivery carriers.^[^ ^27^ ^]^ Flow cytometry revealed the specific binding of aRLP to NEs in the bloodstream. Immunostaining suggested that the proximity of NEs and macrophages facilitated the endocytosis of RLP by the latter. Through the utilization of CM‐specific mCherry mice, it was observed that aRLP treatment disrupted the CD47‐SIRPα axis and facilitated the efferocytosis of CMs by macrophages, leading to improved cardiac remodeling. In addition, taking advantage of the multidrug‐loading potency, a wide variety of drugs could be loaded into nanoparticles which could have a synergistic effect with immune checkpoint inhibition for the treatment of other diseases.

One advantage of TPD is its catalytic activity, which facilitates the continuous degradation of POI by recycling LYTACs.^[^ ^9a^ ^]^ In this study, aRLP demonstrated the ability to degrade SIRPα continuously for a duration of at least 24 h. Due to the temporary exitance of apoptotic CMs, the requirement of CD47‐SIRPα blockage is limited to a brief period after AMI, and continuous degradation might cause undesired side effects. This nano‐degrader hitchhiked the receptor‐mediated internalization pathway and degraded SIRPα in lysosome only in one wave. Though it may be difficult for traditional liposomes to stay stable within macrophages and their ability to recycle and bind to SIRPα may present challenges. This strategy might be particularly applicable to specific conditions that require temporal control over CD47‐SIRPα pathway inhibition, such as cardiac repair after AMI and stem cell transplantation. In the case of diseases necessitating prolonged pathway suppression, nanomaterials capable of recycling to bind additional SIRPα copies could be employed to fabricate the nano‐degrader.

There was also a limitation in our work because it only focused on the SIRPα expressed in macrophages, while it was also expressed in other myeloid cells, including monocytes, granulocytes, and dendritic cells.^[^ ^31^ ^]^ Whether these cells would be affected by aRLP, and if so, would favor the therapeutic effects of aRLP. First, if SIRPα level was downregulated in these cells, we figured that their phagocytosis ability would be elevated. Monocytes and granulocytes are phagocytes that significantly contribute to the clearance of debris and apoptotic CMs after AMI. When the SIRPα level was downregulated, we believe that monocytes and granulocytes would engulf more apoptotic CMs and help a lot in cardiac repair. It has been reported that phagocytosis of apoptotic cells by dendritic cells induces immune tolerance through the generation of Treg cells,^[^ ^32^ ^]^ which might also benefit the prognosis of AMI. Altogether, although the effect of aRLP on monocytes, granulocytes, and dendritic cells was not investigated here, we thought it would contribute to a therapeutic effect, and this remains to be explored further.

## 4 Conclusion

In this study, we fabricated a biomimetic nano‐degrader based on Ly6G antibody‐modified CD47 density‐controlled senescent RBC‐mimetic liposome (aRLP) to promote SIRPα degradation and CD47‐SIRPα immune checkpoint inhibition. By mimicking CD47 density on senescent RBCs, aRLP maintained its ability to bind with SIRPα while disabling its signaling activity. Following SIRPα binding, aRLP facilitated protein degradation by directing it into the lysosome via receptor‐mediated endocytosis. The Ly6G antibody was incorporated into the nanoplatform to address the inherent limitations of poor targeting and compromised immune escape by utilizing neutrophils as a means of transportation. In the AMI model, aRLP was anchored to NEs and subsequently released into nearby macrophages in the ischemic myocardium, leading to improved degradation efficiency. As a result, aRLP effectively inhibited the CD47‐SIRPα immune checkpoint, thereby enhancing macrophage efferocytosis and facilitating cardiac repair through the degradation of SIRPα. Collectively, this study provided a protein degradation strategy for CD47‐SIRPα immune checkpoint inhibition based on the biomimetic nanosystem which could efficiently degrade POI and surpass the inherent limitations of traditional TPD methods, with significant potential for clinical translation.

## 5 Experimental Section

### Animals and Cell Lines

Male C57BL/6J mice were purchased from Shanghai Jiesijie Laboratory Animal Co., Ltd (6–8 weeks old). Animal experiments were approved by the Ethics Committee of Zhongshan Hospital, Shanghai, China (201697). Raw264.7 and HUVEC cell lines were purchased from the Institute of Biochemistry and Cell Biology, Chinese Academy of Sciences (Shanghai, China).

### Induction of Myocardial Infarction Injury

Briefly, mice (8–10 weeks) were anesthetized with isoflurane, and the left anterior descending coronary artery was ligated with a 6‐0 silk suture. After 6 h of ligation, mice were randomized to receive an intravenous injection of PBS, LP, RMV, RLP, and aRLP.

### Primary Adult Cardiomyocyte Isolation and Apoptosis Induction

Primary adult CM isolation was performed as previously described.^[^ ^28^ ^]^ For the in vitro efferocytosis assay, laminin was not added to the CM culture for further transfer. For apoptosis induction, CMs were subjected ischemic buffer (118 mmol L^−1^ NaCl, 24 mmol L^−1^ NaHCO_3_, 1.0 mmol L^−1^ NaH_2_PO_4_, 2.5 mmol L^−1^ CaCl_2_‐2H_2_O, 1.2 mmol L^−1^ MgCl_2_, 20 mmol L^−1^ sodium lactate, 16 mmol L^−1^ KCl, and 10 mmol L^−1^ 2‐deoxyglucose; pH 6.2) and 1% hypoxia for 45 min, which was named oxygen‐glucose deprivation (OGD). For efferocytosis assay, 1 × 10^6^ macrophages were added with 2 × 10^5^ apoptotic CMs for 90 min.

### RBC Ghost Derivation

RBC ghost derivation was done as previously reported.^[^ ^29^ ^]^ RBCs were collected from whole blood from C57BL/6J mice by eyeball extirpating under anesthesia. Specifically, 1 mm EDTA PBS buffer as isotonic solution and 0.25 mm EDTA H2O solution as hypotonic solution were prepared. For each mouse, 200 µL heparin saline was added into 1.5 mL Eppendorf micro test tubes before blood collection to avoid coagulation. Then the whole blood was transferred to 15 mL tubes and 10 mL isotonic solution was added. The whole blood was Centrifugated at 200 g for 5 min at 4 °C (acceleration 9, deceleration 4), and the supernatant was discarded. This process was repeated 3 times. Then 10 mL isotonic solution was added and centrifugated at 2300 g for 5 min at 4 °C. This process was repeated 3 times. Then all the pellet was collected and added to the same volume of isotonic solution. Then every 250 µL solution was added with 950 µL hypotonic solution and vibrated for 10 s. Before centrifugation at 20 000 g for 10 min at 4 °C, 50 µL 20× PBS was added to make the solution isotonic. This process was repeated till the pellet was totally white. Every micro tube of RMV was resuspended with 250 µL hypotonic solution and stored as 150 µL per tube at −80 °C for further use. Thus, every tube equaled 150 µL blood with ≈0.3 mg protein measured by the BCA assay kit (Beyotime, P0012).

### Synthesis of LP, RLP, and aRLP

Conventional liposome (LP) was synthesized by 3.6 mg 1,2‐Dimyristoyl‐sn‐glycero‐3‐Phosphocholine (DMPC), 0.2 mg 1,2‐distearoyl‐sn‐glycero‐3‐phosphoethanolamine‐N‐[methoxy(polyethyleneglycol)−2000] (DSPE‐PEG2000) and 0.2 mg DSPE‐PEG2000‐N3 through film hydration. For nanoparticle tracing, 1,1′‐dioctadecyl‐3,3,3′,3′‐tetramethylindotricarbocyanine perchlorate (DiD) was added (1:1000). The solution was extruded through 0.4, 0.2, and 0.1 µm polycarbonate membranes sequentially (Nuclepore Track‐Etched Membranes, Whatman, UK), using a LiposoFast extruder apparatus (Avestin, Canada) to get final LP.

The 1:1, 1:3, and 1:6 RLP were obtained by extrusion of fixed 0.3 mg protein RMV with 0.3, 0.9, and 1.8 mg LP. Dibenzocyclooctyne (DBCO) modification of Ly6G antibody was performed as previously described.^[^ ^12a^ ^]^ Free DBCO was removed by 10 kDa ultrafiltration centrifugal tube by 4000 g, 40 min at 4 °C. After determination of senescent RBC‐mimetic RLP (1:3), DBCO‐Ly6G was added and shaken at 50 rpm for 1 h to be conjugated to get aRLP. Centrifugation (8000 g) for 10 min at 4 °C of this solution was performed to remove free DBCO‐Ly6G.

### Surface Plasmon Resonance

Real‐time biomolecular interaction analysis was performed using a BiaCore 3000 instrument (GE Healthcare, Piscataway, New Jersey). Mouse Raw264.7 cells were covalently linked at pH 5.0 in 10 mm sodium acetate buffer to a CM5 Chip using EDC/NHS amine coupling of the primary amine of the protein to a carboxyl group of a chip‐linked carboxymethylated dextran using HBS‐P running buffer (10 mm HEPES, 0.15 m NaCl, 0.005% polysorbate 20, pH 7.4) at 25 °C. Ethanolamine was used to block unwanted carboxy groups after linkage (flow rate was 30 µL min^−1^). Background subtraction was based on an ethanolamine‐blocked Fc1. In kinetic determinations, rising concentrations of RLP or RMV (3.75, 7.5, 15, 30, and 50 mg mL^−1^) were measured. After each analyte injection, the chip surface was regenerated with glycine solution (pH 1.5) injection. For affinity constant measurements, replicate injections of each concentration were merged to derive a sensorgram line at each concentration. A Langmuir binding model with local fit (stoichiometry of 1:1) was used to analyze *K*_a_ (association rate constant) and *K*_d_ (dissociation rate constant).

### Immunoprecipitation Assay

Cell lysate (1 mg) was rotated with 2 mg SIRPα antibody (BD Pharmingen, 552 371) for 1 h at 4 °C. Then 20 µL agarose beads (Santa Cruz, SC‐2003) were added overnight at 4 °C. Beads were washed for 3 times at 2500 rpm, 4 °C for 5 min. The final pellet was resuspended in 40 µL loading buffer (2×) at 95 °C for 5 min.

### Primary Neutrophil Isolation

Primary NEs were isolated from mouse bone marrow as previously described^[^ ^21^ ^]^ with modification. Gradient Percoll solution was 81%, 58%, and 45% in PBS (v: v) and fresh NEs were obtained from the interface of 81% and 58%. The purity was identified by flow cytometry and ≈1 × 10^6^ NEs could be isolated from one mouse.

### Migration Ability of aRLP‐NEs

aRLP‐NEs were prepared by incubating 0.3 mg protein aRLP with 2 × 10^5^ NEs and unconjugated aRLP was removed by centrifugation. Human Umbilical Vein Endothelial Cells (HUVECs) were cultured in a cell culture insert (polycarbonate membrane, 3 µm) (Millipore, PIXP01250) for 3 days to form an endothelial layer in vitro. Then 2 × 10^5^ NEs or aRLP‐NEs were added into the upper chamber with or without 10 nM fMLP in the lower chamber. After 3 h, the relative migrated NEs were measured by cell counting kit‐8 (CCK‐8) (Beyotime, C0038).

### Release Profiles of aRLP from aRLP‐NEs

PMA (100 nM) was used in vitro to mimic NETs formation of NEs at the inflammatory site. The mean fluorescence intensity of DiD labeled aRLP was detected at 0, 1, 2, 3, 4, 6, and 8 h time points after PMA stimulation.

### Major organs Distribution

Major organs including the heart, liver, spleen, lung, kidney, and brain, were harvested from mice 1 h after 1.5 mg protein aRLP injection, and nanoparticle signals were imaged by IVIS (PerkinElmer, Inc., Waltham, MA).

### Peripheral Blood Flow Cytometry

Peripheral blood cells were prepared as previously described.^[^ ^14b^ ^]^ Briefly, blood was collected at 0.5, 1, and 6 h after DiD labeled nanoparticle injection and centrifugated at 3000 rpm for 10 min at 4 °C to remove plasma. Then the rest were treated with RBC lysis buffer (Invitrogen, 00‐4333‐57) for 10 min. After centrifugation at 1000 rpm for 5 min and washed with PBS twice, the cells were resuspended and stained with APC‐Cy7‐anti‐CD45 (BD bioscience, 557 659), PerCP‐Cy5.5‐anti‐CD11b (BD bioscience, 550 993), BV421‐anti‐Ly6G (BD bioscience, 562 737), PE‐anti‐Ly6C (BD bioscience, 560 592), and fluorescence signals were detected by BD FACS Aria III.

### Single Cell Suspension of Heart and Flow Cytometry

The heart was digested as previously reported.^[^ ^30^ ^]^ Briefly, the heart was cut into pieces and enzymatically digested with type II collagenase (Worthington, 1.5 mg mL^−1^), Elastase (Worthington, 0.25 mg mL^−1^), DNase I (Worthington, 0.5 mg mL^−1^) for 1 h at 37 °C. After digestion, cells were passed through 70‐µm strainers and stained. For nanoparticle tracing, APC‐Cy7‐anti‐CD45 (BD bioscience, 557659), PerCP‐Cy5.5‐anti‐CD11b (BD bioscience, 550993), BV421‐anti‐Ly6G (BD bioscience, 562737), and AF488‐anti‐F4/80 (BD bioscience, 567201) were used. For macrophage polarization phenotyping, FITC‐anti‐CD45 (BD bioscience, 553080), PerCP‐Cy5.5‐anti‐CD11b (BD bioscience, 550993), PE‐anti‐F4/80 (BD bioscience, 565410), PE‐Cy7‐anti‐CD86 (BD bioscience, 560582), and AF647‐anti‐CD206 (BD bioscience, 565250) were used.

### TTC Staining

Three days post AMI, hearts were harvested from mice and frozen at −40 °C for further cutting. Then the hearts were cross‐sectioned into five slices with 1 mm each and washed with precooled PBS. After incubation in 1% TTC PBS solution at 37 °C for 20 min, 4% paraformaldehyde (PFA) was added to stop the staining and fix the tissue. The slices were scanned by EPSON Perfection V19 and analyzed by Image‐Pro Plus 6.0 (NIH, Bethesda, MD).

### Statistical Analysis

The statistical analysis relied on Student's *t*‐test, one‐way and two‐way analysis of variance (ANOVA) methods using GraphPad Prism (v8.0.1). *p* < 0.05 was considered statistically significant. All data were presented as the mean value ± s.d.

## Conflict of Interest

The authors declare no conflict of interest.

## Author Contributions

J.G., Z.P., and Q.W. contributed equally to this work. J.G., Z.P., and Q.W. performed conceptualization. J.G., Q.W., and Q.L. performed the methodology. J.G., Z.P., Q.W., and Y.T. performed an investigation. H.T., J.C., Y.W., Z.W., H.Y., J.Z., D.S., X.W., Q.W., and J.Q. performed visualization. Y.S., Z.H., and J.G. performed supervision. J.G. wrote the original draft. Y.S. and Z.H. wrote the review and editing.

## Supporting information

Supporting Information

## Data Availability

The data that support the findings of this study are available from the corresponding author upon reasonable request.
