# Supplementary material for: Biomimetic Nano‐Degrader Based CD47‐SIRPα Immune Checkpoint Inhibition Promotes Macrophage Efferocytosis for Cardiac Repair
Source: Adv Sci (Weinh). 2024 Mar 13;11(24):2306388. doi: 10.1002/advs.202306388 (PMC11200091; doi:10.1002/advs.202306388)
Supplement: Supplementary file 1 — Supporting Information [file ADVS-11-2306388-s001.pdf]

ADVANCED SCIENCE
Open Access

Supporting Information

for *Adv. Sci.*, DOI 10.1002/advs.202306388

Biomimetic Nano-Degrader Based CD47-SIRPα Immune Checkpoint Inhibition Promotes Macrophage Efferocytosis for Cardiac Repair

Jinfeng Gao, Zhiqing Pang, Qiaozi Wang, Yiwen Tan, Qiyu Li, Haipeng Tan, Jing Chen, Wusiman Yakufu, Zhengmin Wang, Hongbo Yang, Jinyan Zhang, Dili Sun, Xueyi Weng, Qibing Wang, Juying Qian, Yanan Song, Zheyong Huang* and Junbo Ge**

Biomimetic Nano-degrader Based CD47-SIRPα Immune Checkpoint Inhibition Promotes Macrophage Efferocytosis for Cardiac Repair

Jinfeng Gao [1,2,†], Zhiqing Pang [3,†], Qiaozi Wang [1,2,†], Yiwen Tan [1,2], Qiyu Li [1,2], Haipeng Tan [1,2], Jing Chen [1,2], Wusiman Yakufu [1,2], Zhengmin Wang [1,2], Hongbo Yang [1,2], Jinyan Zhang [1,2], Dili Sun [1,2], Xueyi Weng [1,2], Qibing Wang [1,2], Juying Qian [1,2], Yanan Song [1,2,*], Zheyong Huang [1,2,*], Junbo Ge [1,2,4,*]

[1] *Department of Cardiology, Zhongshan Hospital, Fudan University. Shanghai Institute of Cardiovascular Diseases, Shanghai, 20032, China*

[2] *National Clinical Research Center for Interventional Medicine& Shanghai Clinical Research Center for Interventional Medicine, 180 Feng Lin Road, Shanghai, 200032, China*

[3] *School of Pharmacy, Fudan University, Key Laboratory of Smart Drug Delivery, Ministry of Education, 826 Zhangheng Road, Shanghai, China*

[4] *Institute of Biomedical Sciences, Fudan University, Shanghai, 20032, China*

[*] Corresponding author. Department of Cardiology, Zhongshan Hospital, Fudan University. Shanghai Institute of Cardiovascular Diseases, 180 Feng Lin Road, Shanghai, 200032, China.

E-mail addresses: yanan.song@163.com (Y. Song), zheyonghuang@126.com (Z. Huang), junboge@126.com (J. Ge).

[†] These authors contributed equally.

Calculation of CD47 on RLP

Mouse RBC concentration: 10×10^6 μl^{-1}

Surface area of one RBC: 140 μm^2

Total RBC area of 150 μl blood (0.3 mg protein RMV): $\mathbf{S_1}$=0.21 m^2

One 100-nm LP contains 10^5 PC[1]

3.6 mg PC forms ≈ 2.8×10^{13} LP

Surface area of one 100-nm vesicle: 3.14×10^{-14} m^2

Total surface area of 4 mg LP: $\mathbf{S_2}$= 0.88 m^2

According to 50% CD47 density on senescent RBC, the hybridization ratio of $\mathbf{S_1}$**:**$\mathbf{S_2}$=1:1. That is, 0.3 mg protein RMV and 0.9 mg LP (mass ratio of RMV:LP=1:3).

[1] J. Huwyler, D. F. Wu, W. M. Pardridge, *Proc. Natl. Acad. Sci. U. S. A.* **1996**, 93, 14164.

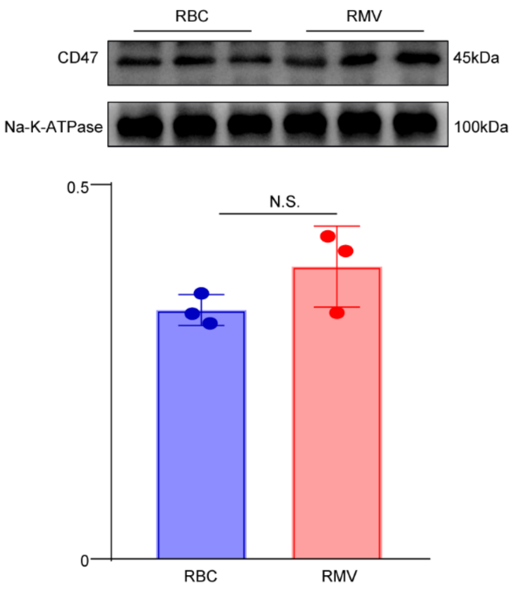

Fig. S1. The relative amount of CD47 protein of RBC and RMV by western blotting and quantification. Statistical analysis was calculated using the two-sided Student's *t*-test (n = 3). Data are presented as mean ± s.d.

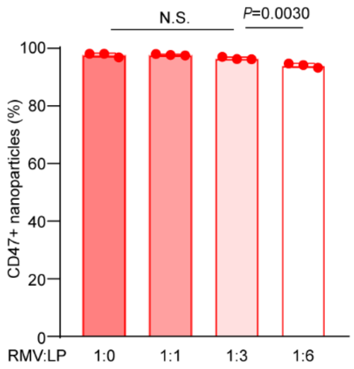

Fig. S2. The CD47 positive nanoparticles of RMV: LP 1:0, 1:1, 1:3 and 1:6. Statistical analysis was calculated using the one-way ANOVA and Tukey's tests (n = 3). Data are presented as mean ± s.d.

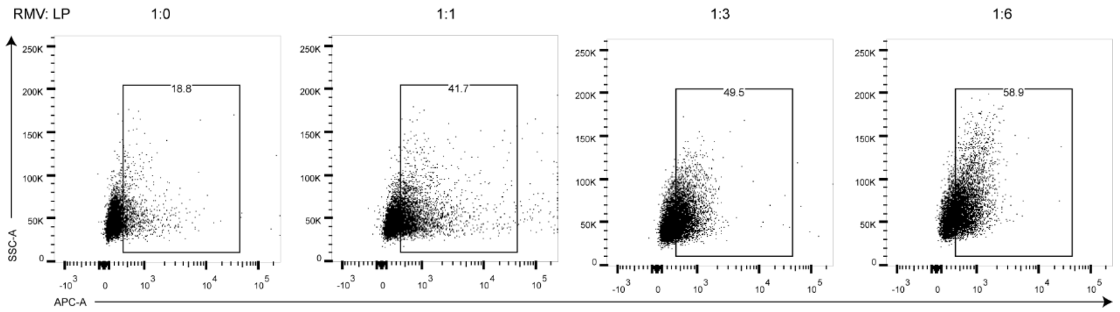

Fig. S3. Representative flow cytometry analysis of RMV: LP 1:0, 1:1, 1:3 and 1:6 particles engulfed by macrophages.

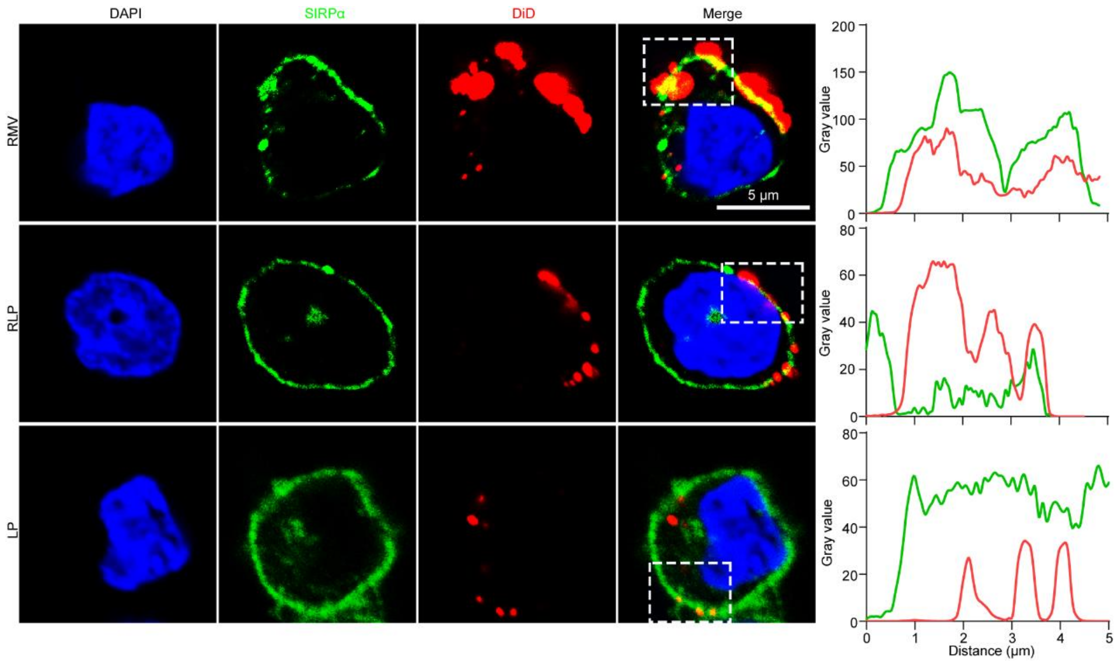

Fig. S4. Representative images of the relative distribution of SIRPα on macrophages contacting with RMV, RLP or LP.

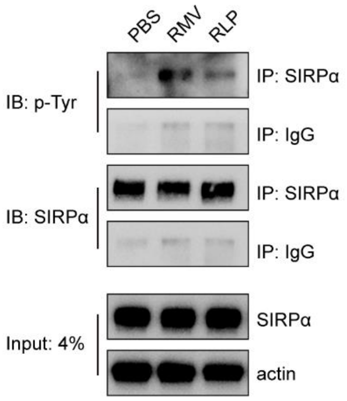

Fig. S5. Immunoprecipitation to detect the SIRPα phosphorylation level of macrophages treated with PBS, RMV or RLP for 20 mins.

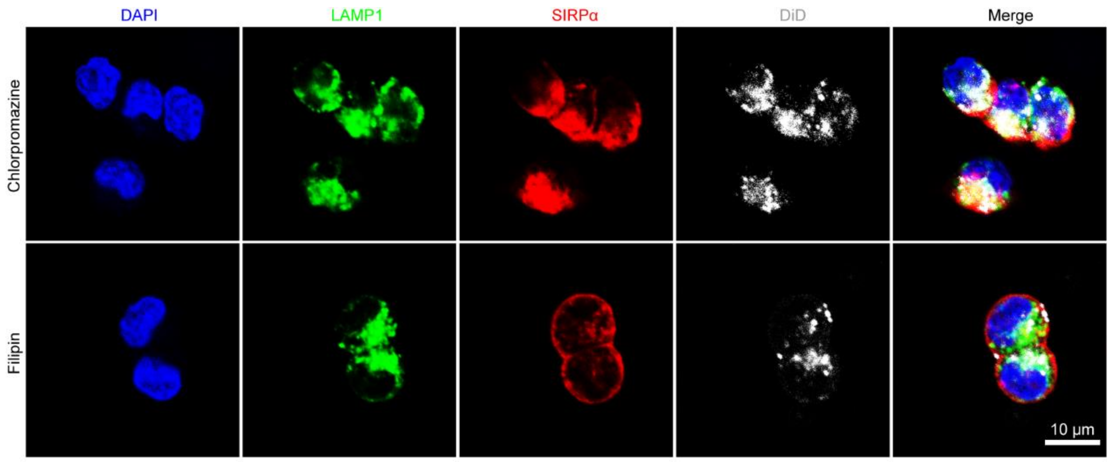

Fig. S6. Representative images of macrophages treated with chlorpromazine (clathrin-mediated endocytosis) and filipin (caveolin-mediated endocytosis) to internalize RLP.

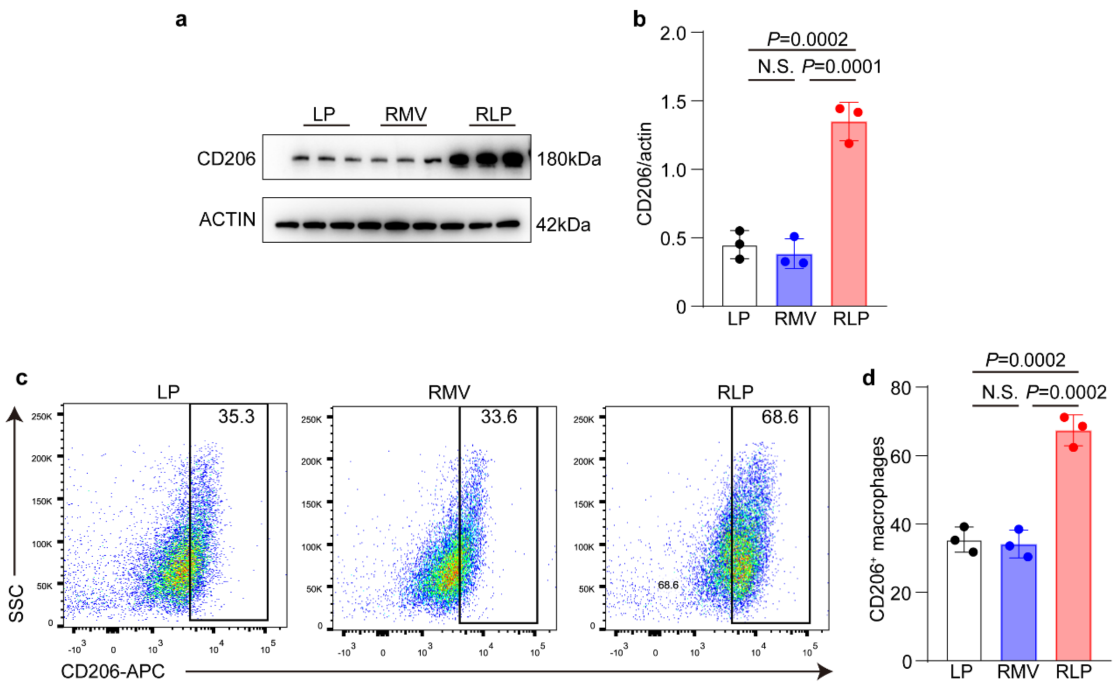

Fig. S7. CD206 expression on macrophages treated with LP, RMV and RLP before efferocytosis. **a, b,** CD206 expression detected by western blotting. **c, d,** CD206 positive macrophages detected by flow cytometry. Statistical analysis was calculated using the one-way ANOVA and Tukey's tests (n = 3). Data are presented as mean ± s.d.

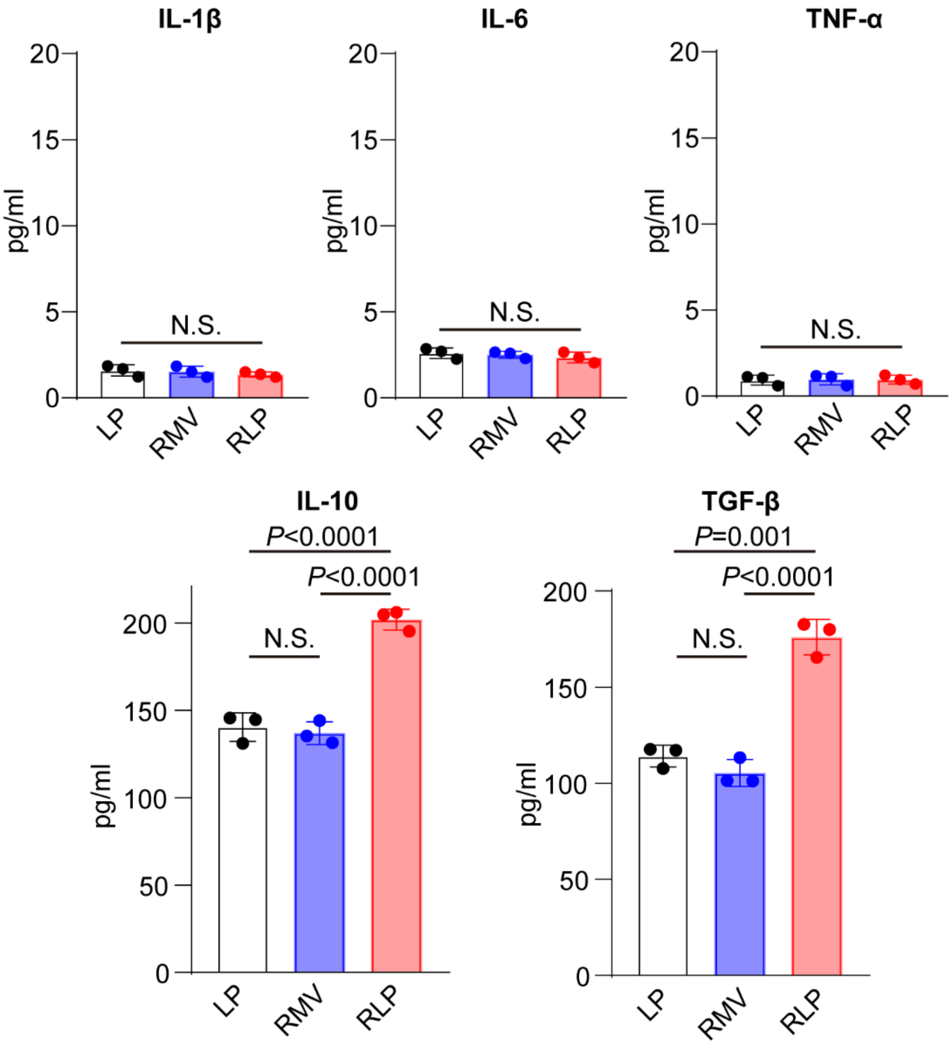

Fig. S8. IL-1β, IL-6 and TNF-α, IL-10 and TGF-β concentration of macrophages supernatants in vitro treated with LP, RMV or RLP before efferocytosis. Statistical analysis was calculated using the one-way ANOVA and Tukey's tests (n = 3). Data are presented as mean ± s.d.

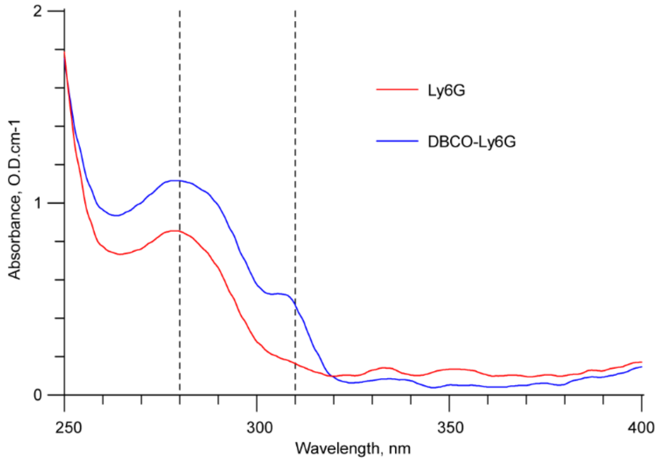

Fig. S9. Ultraviolet-visible spectra of 2.5 μg ml^{-1} unmodified Ly6G and 2.5 μg ml^{-1} DBCO-functionalized Ly6G. The UV absorption band at 310 nm corresponds to absorbance from the conjugated DBCO group.

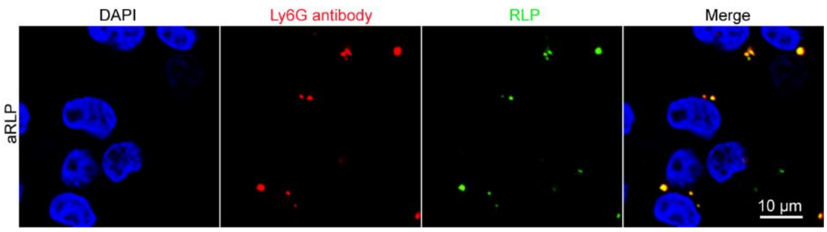

Fig. S10. Colocalization of Ly6G antibody (AF568-goat anti rat) and RLP (DiD) on aRLP incubated with macrophages for 30 min.

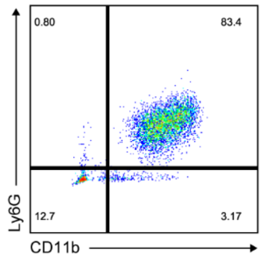

Fig. S11. Purity of primary NEs by flow cytometry.

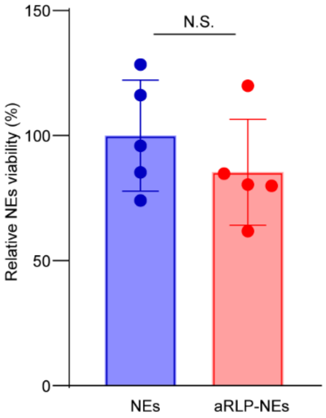

Fig. S12. Relative NEs viability with or without aRLP binding. Statistical analysis was calculated using the two-sided Student's *t*-test (n = 5). Data are presented as mean ± s.d.

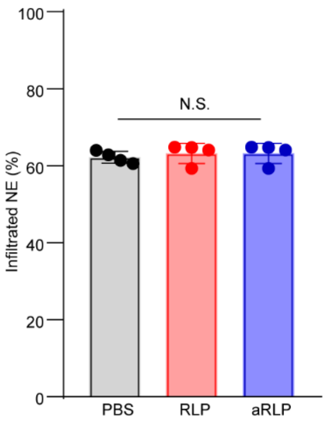

Fig. S13. The percentage of infiltrated NEs in hearts 1 h after injected with PBS, RLP and aRLP. Statistical analysis was calculated using the one-way ANOVA and Tukey's tests (n = 4). Data are presented as mean ± s.d.

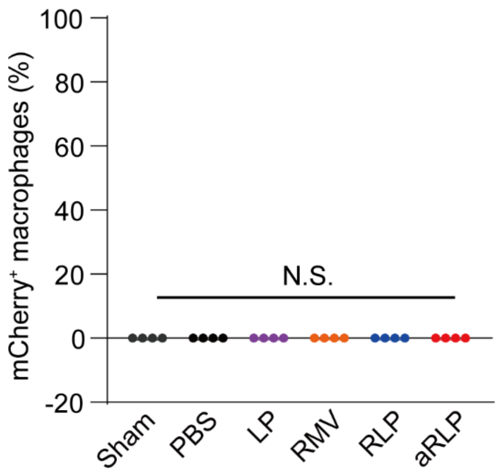

Fig. S14. The efferocytosis efficiency of macrophages in the sham heart or the remote area of infarct heart.

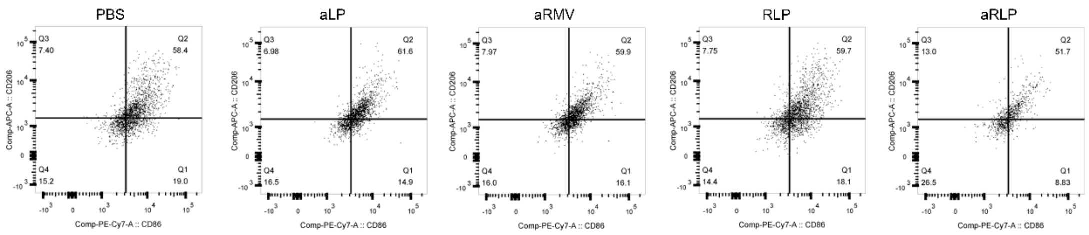

Fig. S15. Representative flow cytometry analysis of heart macrophages after treated with PBS, aLP, aRMV, RLP and aRLP.

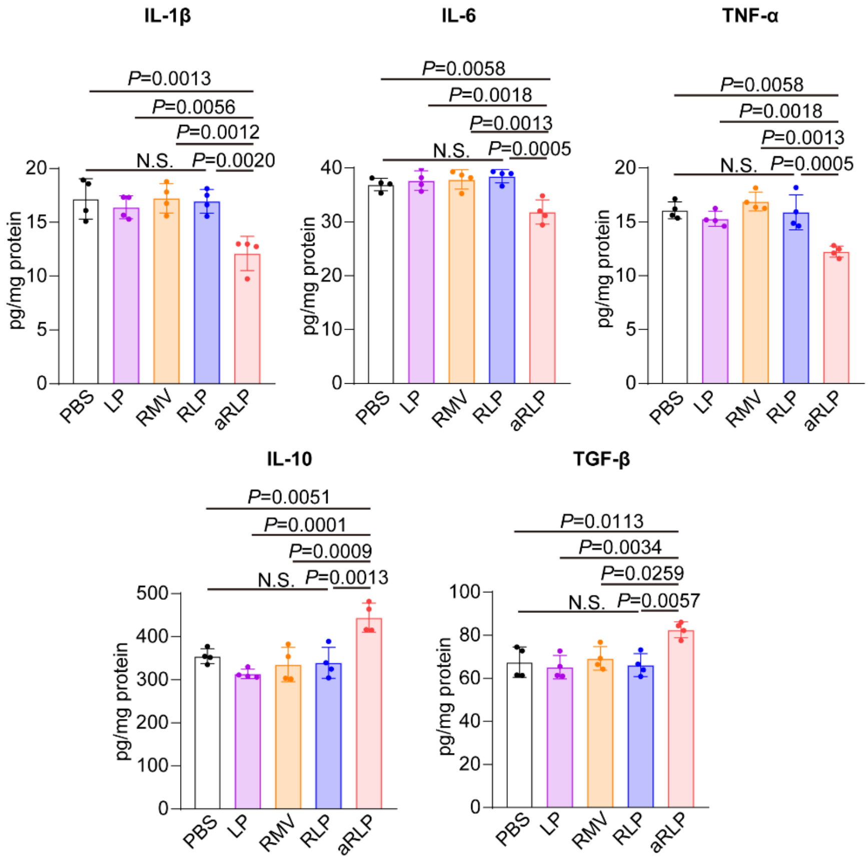

Fig. S16. Concentration of pro-inflammatory mediator IL-1β, IL-6 and TNF-α and anti-inflammatory mediators IL-10 and TGF-β in mouse heart homogenate 3 days after treatment with PBS, LP, RMV, RLP or aRLP. Statistical analysis was calculated using the one-way ANOVA and Tukey's tests (n = 5). Data are presented as mean ± s.d.

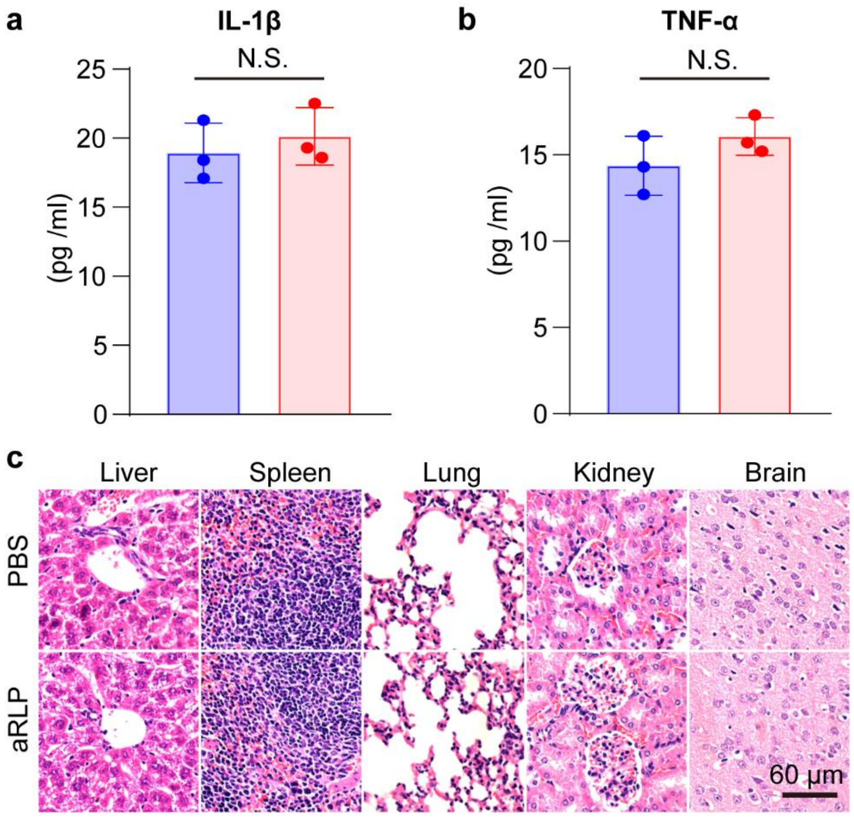

Fig. S17. a, b, IL-1β and TNF-α concentration in plasma 6 hours after PBS or aRLP injection. c, HE staining 7 days after treatment with PBS or aRLP. Statistical analysis was calculated using the two-sided Student's t-test (n = 5). Data are presented as mean ± s.d.

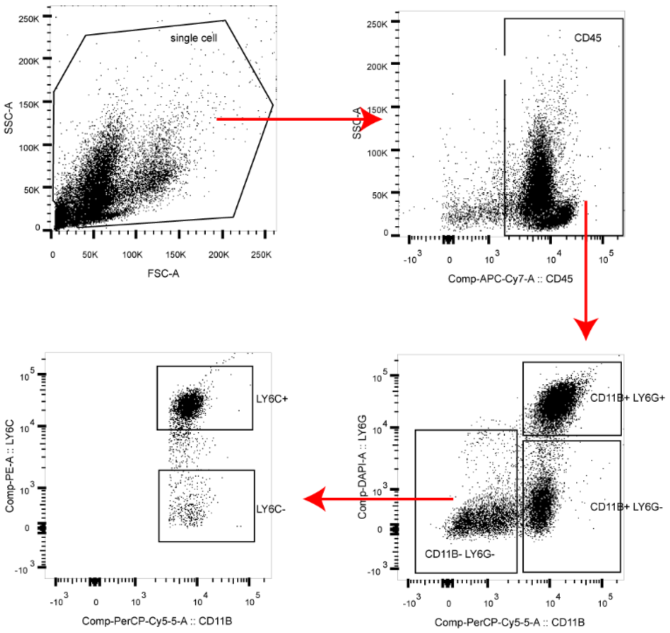

Fig. S18. Gating strategy of flow cytometry analysis of blood cells in Fig. 5b.

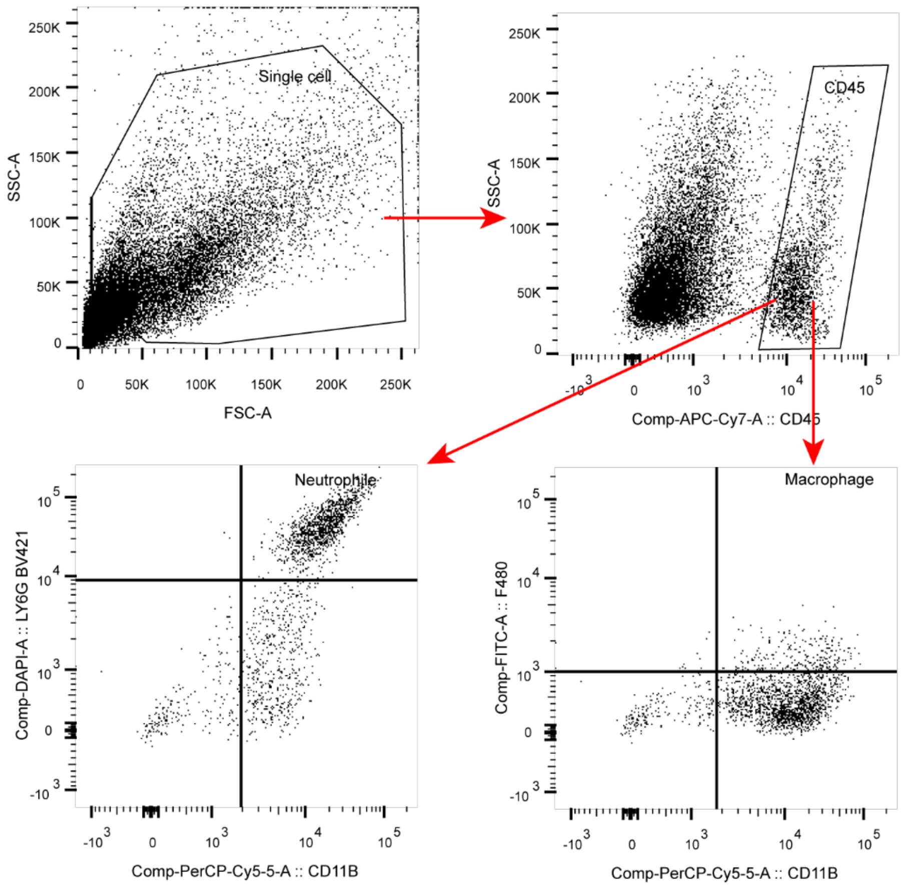

Fig. S19. Gating strategy of flow cytometry analysis of blood cells in Fig. 5e.

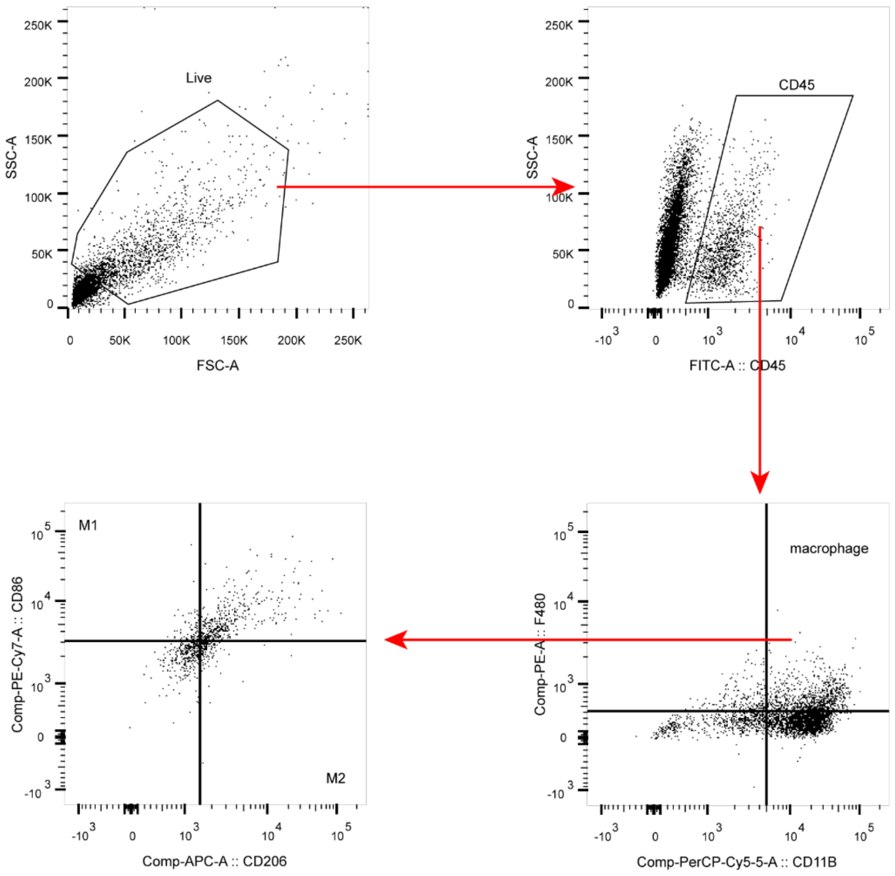

Fig. S20. Gating strategy of flow cytometry analysis of blood cells in Fig. 7g, h.

Table 1. Diameters, PDI and zeta potential of RMV: LP 1:0, 1:1, 1:3, 1:6 and LP.

	1:0	1:1	1:3	1:6	LP
Diameter (nm)	119.8±3.5	122.6±4.8	120.0±4.6	118.6±4.6	120.9±4.6
PDI	0.126±0.007	0.102±0.042	0.099±0.020	0.128±0.033	0.117±0.028
Zeta (mv)	-37.3±1.4	-32.3±0.8	-27.3±1.8	-24.5±1.5	-12.9±1.2

Table 2. Primer sequence for qPCR analysis. RNA isolation was performed with FastPure Cell/Tissue Total RNA Isolation Kit V2 (Vazyme Biotech Co.,Ltd).

Name	Forward	Reverse
actin	GGCTGTATTCCCCTCCATCG	CCAGTTGGTAACAATGCCATGT
IL-1β	GCAACTGTTCCTGAACTCAACT	ATCTTTTGGGGTCCGTCAACT
IL-6	TAGTCCTTCCTACCCCAATTTCC	TTGGTCCTTAGCCACTCCTTC
TNF-α	CCCTCACACTCAGATCATCTTCT	GCTACGACGTGGGCTACAG
IL-10	TTCCCTGGGTGAGAAGCTGA	ATTCATGGCCTTGTAGACACCT
TGF-β	CTGCTGACCCCCACTGATAC	GGGGCTGATCCCGTTGATT
Arg-1	CTCCAAGCCAAAGTCCTTAGAG	AGGAGCTGTCATTAGGGACATC
